# Prevalence and treatment outcome of pulmonary and extrapulmonary pediatric tuberculosis in southwestern Iran 

**Published:** 2015

**Authors:** Seyed Mohammad Alavi, Shokrollah Salmanzadeh, Pejman Bakhtiyariniya, Ali Albagi, Fatemeh Hemmatnia, Leila Alavi

**Affiliations:** 1 Health Research Institute, Infectious and Tropical Disease Research Center, Joundishapur University of Medical Sciences, Ahvaz, Iran; 2 Khuzestan Health Center, Joundishapur University of Medical Sciences, Ahvaz, Iran.; 3 Food and Drug Deputy, Joundishapur University of Medical Sciences, Ahvaz, Iran

**Keywords:** Tuberculosis, Children, Treatment outcomes, Iran

## Abstract

**Background:**

Knowledge about childhood tuberculosis (TB) in Iran is limited. This study aimed to determine the proportion of tuberculosis in children living in Khuzestan in southwest of Iran and its treatment outcomes.

**Methods:**

In this retrospective study, the child’s medical records registered in national TB program (NTP) unit of Khuzestan Health Center (KHC) for TB treatment from 2005 to 2010 were studied. Data including demographic, clinical presentation, laboratory test results, and treatment outcomes were extracted from the files and were analyzed.

**Results:**

Of total 4104 new TB cases registered in KHC, 203 (4.9%) were children. The mean age was 10.7±4.3 years, and 75.7% of them were females. More than 84% of TB children cases were 10 years or older, whereas, young children (< 5 years old) accounted for 5.6%. Of the total studied cases, 57.1% were pulmonary TB and 42.9% were extra pulmonary, 91.7% were successfully treated and 8.3% had poor treatment outcome. The main risk factors for poor treatment outcome were: age <5 years (OR: 0.17, 95% CI, 0.04-0.76), low body weight (OR: 0.08, 95% CI, 0.01-0.60), household contact with cases of TB treatment failure (OR: 0.13, 95% CI, 0.03-0.52), and exposure to cigarette smoke odor inside the home (OR: 0.17, 95% CI, 0.05-0.56).

**Conclusion:**

The proportion of pediatric TB in the region was lower than expected. The treatment success rate was higher than the rate defined in NTP. Special attention should be given to children aged less than five years, low body weight, contact with TB treatment failure cases, and exposure to cigarette smoke.

It is estimated that approximately 1 million cases of tuberculosis (TB) occur in children worldwide, 75% of them occur in the high-burden countries, whereas a low proportion (about 5%) belongs to low-burden countries (1). TB with estimated annual deaths of 74,000 to 130,000 is one of the 10 major causes of mortality among childhood, although the true burden of it in childhood is not well known (1, 2). Tuberculosis is a major public health problem in Iran (3-4). Khuzestan, a southwestern province due to special socioeconomic situation and a long neighboring border with Iraq has a special place in NTP. In a study conducted in Ahvaz, the annual rate of infection was 0.5% and the estimated case of smear-positive TB was approximately 25 per 100000 population. Three children were diagnosed of active TB.

Incidence of pulmonary TB among children in the region regarding to a high prevalence rate of 75 per 100000 population was an interesting subject that could be considered (4). Approximately 6% of all new TB cases in the world belong to children (≤ 15 years), but this ratio is less than 5% in industrialized countries and up to 40% in countries with high prevalence rate of TB (5). 

Previous studies suggested that more than 90% of patients in most short-course regimens for treatment of TB-children had favorable responses. A meta-analysis of studies on TB treatment outcome among children found that, overall the favorable responses were high and death, relapse, and adverse events were uncommon with all regimens used (6) Acquisition of TB in children is usually developed by transmission of TB from adults with active TB. But this issue is not usually considered and so the route of children contamination remains undetected (5).

Increase in the prevalence of tuberculosis among children is an indicator for the failure of national TB control programs (NTP) (1, 2, 5-7). Delay in diagnosis and treatment of sputum smear-positive pulmonary TB (SPPTB) result in continuing transmission of *Mycobacterium tuberculosis* (MT) infection to others especially in children. TB diagnosis in children is difficult because most often they have no sputum, therefore a combination of clinical criteria and non-specific tests are used (7). Delay in TB diagnosis and treatment in childhood, lead to latent TB or high mortality (1, 2, 5). Reactivation of latent TB in next decades (adulthood and elderly) will form infectious reservoir to continue transmission of TB in the community. Monitoring of TB management in children is an essential part of NTP to determine the success or failure treatment outcome. To the best of our knowledge, the status of pediatric TB in Khuzestan has not been addressed yet and so the present study aimed to assess the prevalence rate of TB and to determine the outcome of treatment in children in this province.

## Methods

Children (<15 years) registered as recognized cases of TB over a 6 -year period (2005 to 2010) in Khuzestan Health Center (KHC) were included in the study. Data on demographics, types of TB, microbiological results of sputum smears, HIV status and treatment outcomes were extracted from the patient's medical records provided from 18 cities with a population of approximately 3.5 million. In Iranian health system, program activities against TB are integrated in primary health cares, which are provided by urban or rural health centers fundamentally in primary health units. Diagnosis and treatment of TB cases are made by trained general physician and laboratory experts under the supervision of infectious diseases subspecialist (named TB focal point). In each province, hospital, private clinics and laboratory have to report TB cases to TB units of provincial health centers. Diagnosis and treatment of TB is based on national TB guideline and directly observed treatment strategy (DOTS).

Criteria of TB diagnosis were: positive closed contact history, clinical signs and symptoms, positive chest x-ray (suggesting TB), bacterial evidence; smear or culture (PCR and pathology examination if indicated) and TB skin test. The child was defined as TB case if it had 3 or more of the 5 mentioned criteria (except for positive bacterial evidence in which a positive test is enough for diagnosis). Favorable treatment outcome (cured and completed treatment) and poor outcome (failure, default and death) was measured after 6 months of the standard anti-TB regimen. Other definitions were as follows:

Standard anti-TB regimen or category 1 (Cat 1): 2 months of isoniazid, rifampicin, pyrazinamide and ethambutol (HRZE) and then 4 months of isoniazid and rifampicin (HR).

Cured: A patient whose treatment started after 2-5 months her/his sputum (direct smear) changed from AFB-positive to AFB-negative.

Completed treatment: A patient who completed 6 months of Cat 1, clinically responded to treatment but with unknown bacteriologic status.

Treatment failure: A patient whose treatment started 5 months or more, her/his sputum still remains positive, or within the same time changes from negative to positive again. Or the treatment of smear-negative cases in the beginning, but after two months of treatment, his sputum examination became positive.

Treatment interrupted (default): A patient who is under treatment is said to be defaulted, if he/she left her/his treatment for than 2 months or more.

For calculating treatment outcome, we excluded the patients who were under treatment (at the time of study) and the remaining children with documented treatment outcomes were analyzed. SPSS software was used for data analysis. Chi-square test was used for categorical variables to evaluate the associations between dependent and independent variables. Mann–Whitney test, Fisher’s exact test and multivariate logistic regression were used to analyze the association between variables (e.g. age, sex, pulmonary or extrapulmonary TB, smear positivity, rural or urban residency, body weight, contact with failure TB treatment cases and household smokers) and treatment outcome. The association of predictors with variable was described using 95% confidence interval (CI) and odds ratio (OR). A p-value < 0.05 was considered statistically significant. To evaluate the effect of age on treatment outcome, we divided the patients in two groups below 10 years (as control) and 10 years or more.

## Results

Of the total 4104 new TB cases registered in KHC, 203 (4.9%) were children. From 203 TB children cases, 26 cases were excluded. Finally 177 (87.2%) patients who had complete data in their medical records were enrolled in the study. Among the children with TB, the mean age was 10.7±4.3 years (range 7 months to 15 years), and 75.7% of them were females. The distribution characteristics of pediatric TB cases is shown in table-1.More than 84% of TB pediatric cases were 10 years or older, whereas, young children (< 5 years old) accounted for 5.6%. Of the total 177 cases, 101 (57.1%) were pulmonary TB (PTB) and 76 (42.9%) were extrapulmonary (EPTB). Significant (<0.05) higher proportion of EPTB was seen in children (43%) compared with adults (4%). 

The sites of EPTB are shown in table 1. Among the children with PTB, 73(72.3%) were smear positive (SPPTB) and 27.7% were smear negative (SNPTB). Among the 157 children who had completed the treatment course (after excluding 20 under treatment children) (table 2), 144 (91.7%) were successfully treated and 13(8.3%) had poor treatment outcome (table 3). A total of 144 out of 157 (91.7%) responded to the treatment versus 13 out 157 (8.3%) who did not show treatment response (p<0.05). Young children (<5 years) had a treatment success of 70%, whereas, the rate for treatment success among older children was 93.2% (p<0.05), but there was no significant difference between children below 10 years and older children (table 3).

Only 4 (2.2%) of the registered TB children were documented as dead, 2 cases due to SPPTB (10 years or older), and 2 cases of EPTB (4 years or younger) because of TB meningitis. No cases of co-infected with HIV, DM, immunosuppressive drug use, and drug addiction were observed in studied children.

**Table 1:** Distribution characteristics in children with TB in Khuzestan, 2005-2010

**Variables **	**0-4 years** **N=10**	**5-9 years** **N=18**	**10-15 years** **N=149**	**Total** **N=177**
**Sex** Female Male	3 (30) 7 (70)	9 (50) 9 (50)	122 (81.9) 27 (18.1)	134 (75.7) 43 (24.3)
**PTB** Smear-positive Smear-negative	0 4 (40)	3 (16.7) 10 (55.5)	70 (47.0) 14 (9.4)	73 (41.3) 28 (15.8)
**EPTB** Lymphadenitis Meningitis Miliary Peritonitis Osteoarticular Others	3 (30) 2 (20) 1 (10) 0 0 0	4 (22.2) 0 0 1 (5.5) 0 0	40 (26.8) 2 (1.3) 3 (2.0) 5 (3.4) 7 (4.7) 8 (5.4)	47 (26.5) 4 (2.3) 4 (2.3) 6 (3.4) 7 (3.9) 8 (4.5)

Abbreviation: PTB; pulmonary tuberculosis, EPTB; extra pulmonary tuberculosis

**Table 2:** Treatment outcomes among children with TB in Khuzestan, 2005-2010

**Variables **	** Treatment outcome**				
	**Cured** **(N=56)**	**Completed treatment** **(N=88)**	**Deceased** **(N=4)**	**Failure** **(N=1)**	**Defaulted** **(N=8)**
**Age (years)** 0-4 5-9 10-15	0 1 (1.7) 55 (98.2)	7 (8.0) 13 (14.7) 68 (77.3)	2 (50) 0 2 (50)	0 0 0	1 (12.5) 0 7 (87.5)
**Sex ** Female Male	47 (83.9) 9 (16.1)	48 (54.5) 40 (45.5)	2 (50) 2 (50)	0 0	5 (62.5) 3 (37.5)
**PTB** SPPTB SNPTB EPTB	56 (100) 0 0	5 (5.7) 28 (31.8) 55 (62.5)	2 (50) 0 2 (50)	1(100) 0 0	1 (12.5) 3 (37.5) 4 (50)

Abbreviation: PTB; pulmonary tuberculosis, EPTB; extrapulmonary tuberculosis, SPPTB; smear-positive pulmonary tuberculosis, SNPTB; smear-negative pulmonary tuberculosis

**Table 3:** Comparison of treatment outcome in pediatric children with tuberculosis in Khuzestan, a southern province of Iran with calculated odds ratio (OR) with corresponding 95% confidence interval (95%CI) (2005-2010)

**Variables **	**Successful outcome** **N=144**	**Poor outcome** **N=13**	**OR, 95%CI**	**P-value**
Age (years) <10 ≥10	21 (14.6) 123 (85.4)	3 (23.1) 10 (76.9)	0.57,0.14-2.24	0.42
Sex Female Male	95 (66.0) 49 (34.0)	8 (61.5) 5 (38.5)	1.94,0.54-7.02	0.42
PTB EPTB	89 (61.8) 55 (38.2)	7 (53.8) 6 (46.2)	1.39,0.44-4.34	0.36
SPPTB SNPTB	61 (42.4) 28 (19.4)	4 (30.8) 3 (23.1)	1.63,0.34-7.79	0.22
Rural Urban	51 (35.4) 93 (64.6)	2 (15.4) 11 (84.6)	3.02,0.64-14.14	0.61
Low body weight*	2 (1.4)	2 (15.4)	0.08,0.01-0.60	0.005
Contact with failure TB*	8 (5.6)	4 (30.8)	0.13,0.03-0.52	0.02
Exposure to household *smoker	24 (16.7)	7 (53.8)	0.17,0.05-0.56	0.004

Abbreviation: PTB; pulmonary tuberculosis, EPTB; extrapulmonary tuberculosis, SPPTB; smear-positive pulmonary tuberculosis, SNPTB; smear-negative pulmonary tuberculosis, CI; confidence interval, OR; odds ratio,

*; statistically significant.

## Discussion

The present study revealed that children contributed to 4.9% of the total TB patients registered by NTP surveillance system in the province, older children (10–14 years) represented 84.2% of them. Age and sex in male and female young children (0-4 years) were equally distributed, whereas, in older children(10-15 years) females were affected more than males, (57.1%) pulmonary TB and (42.9%) extra pulmonary .The majority (72.3%) of children with PTB were smear positive, and 91.7% of patients responded to the treatment. The proportion of pediatric TB in our study is consistent with the results of studies performed in developed countries (1, 2, 8), whereas, this proportion is lower than that in high TB incidence area (such as the less developed countries) where this rate is estimated to be up to 40% (1, 2, 7-11). 

We do not believe that the low incidence of tuberculosis in children in Khuzestan is similar to industrialized countries but, this may be due to negligence of pediatric TB in the province. According to NTP, case finding of TB in children as well as adults is passively provided, therefore, many child TB cases may be misdiagnosed due to atypical presentation (12). Difficulties in TB diagnosis in children are the most important challenges in the way of control programs in most countries, even in developed countries. Alavi et al. in a study in Khuzestan showed that the actual incidence of TB in children is higher than those reported through the current passive case finding (4). 

Young children have a higher risk of progression to active TB (13). In previous studies, under-five children constituted the majority of childhood TB (8, 10, 14, 15). Hailu et al. reported a proportion of 48.2% in young children (10). In our study, older children represented the majority of pediatric TB cases. The reason for this difference in our results with other studies is not clear to us but we believe that it is probable in our study, TB among young children may have been prevented.

In our study, male and female young children are equally affected with TB, whereas in older children females are affected more than males. This finding is consistent with most studies which were already conducted (10, 16). In the present study, the majority of children with PTB were smear positive which is opposite with previous reports (10). This difference is probably because most children in our study were older than 10 years and had cavities in imaging studies (secondary TB), whereas most patients in Hailu's study presented with primary rather than secondary and therefore, were likely to be paucibacillary. Young children also do not produce sputum; therefore their diagnosis include the clinical criteria and chest x-ray evidence.

The proportion of children with EPTB (43%) in the current study is similar to previous studies from African countries; with results ranged from 40% to 47% (10, 16, 17). With regard to the epidemiology of tuberculosis in Iran and in comparison with high burden of TB countries, it seems that the high amount of EPTB in our children may be caused by overdiagnosis, and therefore, reconsideration should be made in Iran's national TB program.

In this study, 91.7% of patients responded to the treatment, this rate was higher than the rate (87%) determined in NTP, and recommended by WHO (18). The rate of TB treatment responders among children in our study is lower than the rate (95%) in India (11), but significantly higher than the rate of 66.7% from other geographic regions of Iran (19), and the rate (85.5%) reported was from different countries (10, 15). These differences may be due to differences in economic, social, cultural, geographic, health care facilities, incidence of TB in the region, microbial resistance, HIV prevalence in the country, rate of intravenous drug users, and parental awareness of TB. The mortality rate (2.2%) in our study is nearly similar to the mortality rate (3.3%) reported by Hailu et al. (10) but lower than the rate (7.1%) reported by Salarri et al. (19).The reason for the low mortality rate in our region compared to Yazd province (with a better socioeconomic and cultural status) is not clear but, we believe that the lack of data on the treatment outcomes of patients with incomplete data were excluded in which mortality could be higher that might have resulted in the underestimation of the mortality rate.

 In the present study, both smear positive and smear negative PTB cases had similar response to treatment. In contrast to this finding, some investigators reported that having smear positive PTB is associated with favorable response to treatment (11), whereas, some others reported that smear positive PTB is associated with poor treatment outcomes (10). However, it may be concluded that children with smear positive PTB because of advanced cavitary disease are at the risk of failure or death.

In our study, low body weight (indicator of malnutrition), household contact with TB treatment failure, and household exposure to cigarette smoke were the main risk factors for poor response to anti-TB therapy. The poor treatment outcome in children less than 10 years was similar to older children. This finding is in contrast with previous studies; Hailu et al. in Ethiopia (10), Salarri et al. in Iran (19), and Wu et al. in China (20). The reason for this difference may be due to comparing the younger than 10 years children with older ones. It seems young children (younger than 5 years) due to immature immune systems are at increased risk for disseminated TB and TB meningitis with a higher mortality and poor treatment outcome (10, 19, 20).

In our study, a child who is a household or close contact of a treatment failure index case had poor response to treatment. We did not investigate drug susceptibility among children in this study but, the fact that the risk of multidrug-resistant tuberculosis in patients with retreatment is about twenty percent, thus, the risk of multidrug-resistant tuberculosis in children in household contact with these patients may increase. We did not find any research to present malnutrition and exposure to smokers as risk factors for poor treatment outcome among children with TB; hence, we cannot compare our findings with other studies. It is documented as: cigarette smoke and malnutrition reduce local and systemic defense and are known risk factors for tuberculosis which may have the reverse effect on TB treatment outcome (8).

There were some limitations in this study. This kind of study was retrospective; so, we had no access to useful data such as, family socioeconomics, parents’ behavior and their knowledge about TB. We had no data about the treatment outcome of patients transfer-out and transfer-in under treatment patients. Excluding them from our calculation may be the reason for some biases in mortality or poor treatment outcome. This study has limitations in its lack of data for patient compliance with medication. It is possible that no responders took anti-TB drugs appropriately as recommended. In addition, sensitivity of MT to available groups has not been assessed and so responders and non- responders may had different MT and required different regimens.

**Conclusion**: The proportion of pediatric TB in the region is lower than expected. The treatment success rate was higher than the rate defined in NTP. Special attention should be given to children having low body weight, contact with failure treatment TB cases and exposure to cigarette smoke.

## Notes

**Financial support:** This study was supported by the Infectious and Tropical Research Center of Ahvaz, Joundishapur University of Medical Sciences.
